# *Tropical Medicine and Infectious Disease* John M Goldsmid Award 2018

**DOI:** 10.3390/tropicalmed4010019

**Published:** 2019-01-26

**Authors:** TropicalMed Editorial Office

**Affiliations:** MDPI, St. Alban-Anlage 66, 4052 Basel, Switzerland

For many years, The Australasian College of Tropical Medicine (ACTM) has awarded the John M Goldsmid Award for the best paper published in its official journal, the *Annals of the ACTM,* in the previous year. The Award had been established in honor of Emeritus Professor John M Goldsmid, who was the Inaugural Editor of the Annals, from 2002–2008. He was also ACTM President 1998–2000 and was elected as an Honorary Fellow of the College in 2001. The award now continues to be presented in the College's new official publication, *Tropical Medicine and Infectious Disease*. 

We are pleased to announce the “John M Goldsmid Award” for 2018. Nominations, chosen from all papers published in 2016–2017, were made by the Editorial Board. Following review by the Editorial Board, the three top-voted articles as follows, have each won the “John M Goldsmid Award” for 2018:

## 1st Prize

**Amal K. Mitra and Anthony R. Mawson**

Neglected Tropical Diseases: Epidemiology and Global Burden

*Trop. Med. Infect. Dis.***2017**, *2*, 36. doi:10.3390/tropicalmed2030036

https://www.mdpi.com/2414-6366/2/3/36

## Runner Up Awards

**Sarah L. Smiley, Andrew Curtis, and Joseph P. Kiwango**

Using Spatial Video to Analyze and Map the Water-Fetching Path in Challenging Environments: A Case Study of Dar es Salaam, Tanzania 

*Trop. Med. Infect. Dis.***2017**, *2*, 8; doi:10.3390/tropicalmed2020008

https://www.mdpi.com/2414-6366/2/2/8

## Arnaud Tarantola

Four Thousand Years of Concepts Relating to Rabies in Animals and Humans, Its Prevention and Its Cure 

*Trop. Med. Infect. Dis.***2017**, *2*, 5; doi:10.3390/tropicalmed2020005

https://www.mdpi.com/2414-6366/2/2/5

We believe that these three exceptional papers make valuable contributions to *Tropical Medicine and Infectious Disease* and the scientific research field. On behalf of the *Tropical Medicine and Infectious Disease* Editorial Board and the College, we would like to congratulate these teams for their excellent work. The winners of the 1st prize will receive a cash honorarium of 500 CHF. All the authors will be each offered the privilege of publishing a paper free of charge in open access format in *Tropical Medicine and Infectious Disease*, after the usual peer-review procedure. A certificate will be given to each of the Award recipients.

We would like to take this opportunity to thank all the nominated research groups of the above exceptional papers for their contributions to *Tropical Medicine and Infectious Disease*, and to thank the *Tropical Medicine and Infectious Disease* Editorial Board for voting and helping with this “John M Goldsmid Award”.

The Editorial Board and Editorial Staff at *Tropical Medicine and Infectious Disease* are committed to meeting the needs of the science community by providing useful and timely reviews of all manuscripts submitted and providing an open access journal for your results. Please consider submitting your work to *Tropical Medicine and Infectious Disease*, and we look forward to announcing your paper as a *Tropical Medicine and Infectious Disease* Best Paper in the future.

## John M Goldsmid Award Committee

*Tropical Medicine and Infectious Disease* Editorial Board

